# Shaoyao-Gancao Decoction Ameliorates the Inflammation State in Polycystic Ovary Syndrome Rats via Remodeling Gut Microbiota and Suppressing the TLR4/NF-κB Pathway

**DOI:** 10.3389/fphar.2021.670054

**Published:** 2021-05-13

**Authors:** Zhuang-peng Chang, Gui-feng Deng, Yun-yun Shao, Ding Xu, Yi-nan Zhao, Yi-fan Sun, Shi-quan Zhang, Rui-gang Hou, Jun-jin Liu

**Affiliations:** ^1^ School of Pharmaceutical, Shanxi Medical University, Taiyuan, China; ^2^ Department of Pharmacy, Second Hospital of Shanxi Medical University, Taiyuan, China

**Keywords:** polycystic ovary syndrome, shaoyao-gancao decoction, gut microbiota, gut barrier, TLR4/NF-κB pathway

## Abstract

**Background:** Emerging evidence suggests that gut microbiota plays a vital role in the occurrence of multiple endocrine disorders including polycystic ovary syndrome (PCOS). Shaoyao-Gancao Decoction (SGD), a classical Chinese prescription, has been widely used in the treatment of PCOS for decades. In previous studies, we found that SGD treatment could effectively reduce ovarian inflammation in PCOS rats. However, whether the anti-inflammation effect of SGD involves the regulation of the gut microbiota remains elusive.

**Methods:** Letrozole-induced PCOS rat models were established, and the therapeutic effects of SGD were evaluated. Specifically, body weight, serum hormone concentrations, estrus phase and ovary histopathology were assessed. Then the structure of gut microbiota was determined by 16s rRNA sequencing. Additionally, the serum levels of pro-inflammatory cytokines and LPS were measured by ELISA kits. The key gene and protein expressions of TLR4/NF-κB signaling pathway were detected by quantitative real-time PCR and western blot.

**Results:** SGD could effectively reduce body weight, regulate estrous cycles and ameliorate hyperandrogenism in PCOS rats. In addition, SGD treatment decreased releases of pro-inflammatory cytokines, enhanced the expressions of tight junction (occludin and claudin1), and then prevented a translocation of LPS into bloodstream. SGD could significantly reduce the ratio of *Firmicutes* to *Bacteroidetes*, decrease the abundance of LPS-producing pathogens *Proteobateria* and enrich the abundance of *Butyricicoccus, Coprococcus, Akkermansia Blautia* and *Bacteroides* in PCOS rats. Furthermore, SGD blunted the key gene and protein expressions of TLR4/NF-κB signaling pathway both *in vivo* and in LPS-induced RAW264.7 cells.

**Conclusion:** SGD administration could ameliorate the inflammatory response in PCOS rats by remodeling gut microbiome structure, protecting gut barrier, and suppressing TLR4/NF-κB signaling pathway.

## Introduction

Polycystic ovary syndrome (PCOS) is a complex hormonal imbalance disease that affects 5–20% reproductive women and causes high rate of infertility (de Medeiros et al., 2018). Clinically, the main characteristics of PCOS are hyperandrogenism, hirsutism, acne, menstrual disorders and infertility. Although the common onset age for PCOS is adolescence, the accompanying complications (such as insulin resistance, cardiovascular disease and obesity) occurred throughout the women’s whole life span (Anagnostis et al., 2018). Nowadays, numerous studies have suggested that the low-grade chronic inflammatory status of PCOS was inseparably associated with these clinical features and the long-term metabolic disorders (Rostamtabar et al., 2021). Typically, females with PCOS are predisposed to have elevated levels of pro-inflammatory cytokines [e.g., interleukins (IL)-6 and IL-18] as well as the declined levels anti-inflammatory cytokines (e.g. IL-10 and IL-22) (Qi et al., 2019; Wang et al., 2020a). The imbalanced inflammatory state in PCOS ovaries could not only discount the insulin sensitivity but also impair the follicular growth. Therefore, chronic inflammation has been increasingly recognized as a cornerstone of PCOS pathology.

Recently, accumulating researchers realized that the gut dysbacteriosis is inextricably linked to the onset of the chronic inflammatory diseases, especially PCOS (Insenser et al., 2018; Torres et al., 2018). A large number of animal studies have confirmed the alternation of gut microbiome in PCOS, including a decline in α diversity and the alternation in β diversity (Wang et al., 2020c; Zhu et al., 2020). In clinical studies, females with PCOS also shown extremely different intestinal microbiome compared to healthy controls (Eyupoglu et al., 2020). According to “the Dysbiosis of Gut Microbiota theory”, which was first proposed by Tremellen and Pearc, the change of gut microbiota could result in the activation of the host’s immune system and initiate a state of low grade inflammation in PCOS (Tremellen and Pearce, 2012). Hence, maintaining the homeostasis of gut microbiota provides us a new insight in explaining the etiology behind PCOS.

Generally, the homeostasis of gut microbiota is closely associated with the gut barrier integrity. When the normal microbiology was disturbed, the gut mucosal barrier was damaged and caused the “leaky gut”. As a result, the bacterial endotoxin such as lipopolysaccharide (LPS) leak from the gut lumen into the systemic circulation. LPS, an important product of gut Gram-negative microbiota, is a natural ligand of toll-like receptor 4 (TLR4). Upon LPS recognition, TLR4 recruits a sequence of downstream adaptors and then triggers the signaling cascade. Thereafter, the downstream mediators [such as nuclear factor-κB (NF-κB) and phosphatidylinositol 3-kinase (PI3K)] were activated and numerous pro-inflammatory cytokines (e.g., tumor necrosis factor (TNF)-α, IL-1β and IL-18) were released consequently (Liu et al., 2010). Of note, the elevated serum level of LPS had been observed in women with PCOS and the index was regarded as a risk factor of PCOS (Banaszewska et al., 2020). The LPS-induced inflammatory response occurs in multiple tissues including ovarian theca. As reported, the activation of TLR4/NF-κB signaling contributes to the ovulatory disruption by creating a pro-inflammatory environment in ovary (Wang et al., 2020a). Therefore, effective improve of the microbiota-driven inflammatory state could be a promising avenue to ameliorate PCOS.

Originated from the Treatise on Febrile diseases (a classical work written by Zhongjing Zhang), Shaoyao-Gancao Decoction (SGD), a traditional Chinese herbal prescription has been widely used to treat multiple gynecological disorders such as dysmenorrheal, adenomyosis and PCOS. According to Chinese medical classics, SGD contains of *Paeonia lactiflora Pall* (also known as peony) and *Glycyrrhiza uralensis Fisch. ex DC* (also known as licorice) in the ratio of 1:1*.* Previously, we found that SGD could dose-dependently reduce body weight, alleviate chronic inflammation and rebalance the serum hormonal levels in PCOS rats (Shao et al., 2019). The excellent therapeutic effect arouses our interest to explore the further mechanism. Recent lines of evidence have shown that the active constituents of peony and licorice could effectively improve the gut microbial dysbiosis. For instance, the total glucosides of peony were reported to augment the abundance of beneficial bacteria in the gut (Luo et al., 2019). However, whether the decoction could remodel the intestinal homeostasis has not yet been reported. Hence, the aim of this study was to clarify whether SGD administration could remodel the composition of gut microbiota and thereby alleviate the symptoms of PCOS. Furthermore, we also explore whether the underlying mechanism is relevant to the TLR4/NF-κB signaling pathway. This study could help to better understand the potential mechanisms of SGD in therapy of PCOS from the perspective of gut microbiota.

## Materials and Methods

### Chemicals and Reagents

Letrozole Tablets (2.5°mg/piece) were purchased from Hengrui Medicine Co., Ltd. (Jiangsu, China). ELISA kits were purchased from Sinobestbio (Shanghai, China). Primary antibody TLR4 (1:1000, GB11519), p65 (1:1000, GB11997), phosphorylated (p)-p65 (1:000, GB13025-1), Akt (1:1000, GB111114), GAPDH (1:2000, GB11002), and horseradish peroxidase (HRP)-conjugated secondary antibody (1:3000, GB23303) were purchased from Servicebio (Wuhan, China). Primary antibody PI3K (1:1000, A18640), p-PI3K (1:1000, AP0854), and p-Akt (1:1000, AP0637) were purchased from ABclonal (Wuhan, China). RIPA lysis buffer were purchased from Boster (Wuhan, China). RNAprep pure Tissue/Cell Kit, FastKing RT Kit, and SuperReal PreMix Color (SYBR green) were purchased from TIANGEN (Beijing, China). Dulbecco’s modified Eagle's medium (DMEM), fetal bovine serum (FBS), penicillin-streptomycin were purchased from Gibco (USA). NO assay kit and cell counting kit-8 (CCK-8) were purchased from Elabscience Biotechnology Co., Ltd. (Wuhan, China). Mag-Bind Soil DNA Kit was purchased from Omega Bio-Tek (United States).

### Animals and Induction of PCOS

Female Sprague Dawley (SD) rats (180 ± 20 g) were purchased from the Laboratory Animal Center of Chinese Food and Drug Administratory (Beijing, China). The rats were housed in the animal facility with a temperature of 23 ± 2°C, a relative humidity of 50 ± 10%, and a 12 h light/dark cycle for 1°week prior to the experiment. They had free access to standard food and water ad libitum. The animal study was reviewed and approved by Ethics Committee of the Second Hospital of Shanxi Medical University (License: 2015KS001).

After 1°week of acclimatization, the PCOS rats model was induced as described previously (Liu et al., 2019). In brief, animals were orally administrated daily with 1 mg/kg letrozole for 21 consecutive days. The rats in the normal group were given the same volume of 0.5% CMC-Na aqueous solution. From the first day after modeling, the estrous cycles of rats were observed by performing a vaginal smear. The rats with a disordered estrus cycle during modeling period were considered to be PCOS rats.

### Plant Material and SGD Preparation

*Paeonia lactiflora Pall* (Anhui, China) and *Glycyrrhiza uralensis Fisch. ex DC* (Inner Mongolia, China) were purchased from Hebei Lerentang Medicine Co., Ltd. (Hebei, China). The plants were identified by Professor Jing-ping Zhang (Department of Pharmacy, Second Hospital of Shanxi Medical University, Taiyuan, China). The SGD were prepared as described by Shao et al. Briefly, the *Paeonia lactiflora Pall* (1°kg) and *Glycyrrhiza uralensis Fisch. ex DC* (1°kg) were mixed together and pulverized into powder, and subsequently were macerated using distilled water (1:10, w/v) for 1 h and boiled twice (1 h each time). Afterwards, the liquids obtained were blended, filtered, and concentrated to 4.24 g crude material per ml. As previously described, seven active components were used as the quantity control of SGD and were analyzed by ABI 5500 QTRAP mass spectrometer (Shao et al., 2020b). The content of main active ingredients such as glycyrrhizic acid, paeoniflorin, liquiritin, albiflorin, liquiritigenin, oxypaeoniflorin and glycyrrhetinic acid per 25 g SGD is about 88.50, 48.29, 21.34, 8.25, 3.71, 0.59 and 0.35 mg respectively, which meet the reasonable dose range for *in vivo* studies (Heinrich et al., 2020). The chromatogram was shown in Supplementary Figure S1.

### Experimental Design

To investigate the therapeutic effects of SGD on PCOS, rats were randomly divided into three groups (*n* = 10 per group): normal group, model group and SGD group. Our previous study showed that treatment with SGD at 25 g/kg/day can effectively improve the symptoms of PCOS rats (Shao et al., 2019). Therefore, in this experiment, the rats in SGD group were treated with SGD at 25 g/kg/day after successful modeling. Meanwhile, the rats in normal and model groups received equal volumes of physiological saline. The pharmacological intervention was sustained for 2 weeks and the body weight was recorded every day. Finally, all of the rats were anesthetized and sacrificed followed by the blood and both ovarian tissues were collected. The blood samples were obtained for sex hormone level tests. One side of the ovaries in each rat was fixed in 4% paraformaldehyde buffer for histopathological examination, and the other side was stored at −80 C for subsequent real-time quantitative PCR (RT-qPCR) and western blot analysis.

Secondly, to investigate the effect of SGD on gut microbiota in PCOS rats, on the day before the rats were sacrificed, the feces of all rats were collected for 24 h using sterilized equipment and were quickly frozen in liquid nitrogen for 16S rRNA gene sequencing analysis. And then we measured the content of LPS in rat serum and evaluated the protection function of SGD on intestinal mucosal barrier.

Finally, to explore the underlying mechanism of TLR4/NF-κB inflammatory signal pathway participation, the relative gene and protein expressions of the pathway in the ovarian tissues and the downstream inflammatory factor levels in the serum were measured. Furthermore, we validated the molecule mechanism using the cell experiment. LPS was applied to induce the activation of TLR4 and then the expressions of the pathway-related genes were detected.

### The Determination of Estrus Cycle Phases

Vaginal smears of all rats were performed daily to determinate estrus phase during the experiment. Briefly, a sterile cotton swab dipped in saline lightly scraped the cells of the rat vagina wall and smeared it evenly on the glass slide clockwise. The methanol-fixed smears were stained with Giemsa for 20 min. The cells in the smears were examined under a light microscopy at ×100magnification, and the stages in estrous cycles were assessed based on vaginal cytology.

### Histopathological Analysis of Ovarian Tissues

The ovarian tissues fixed in 4% paraformaldehyde buffer were embedded in paraffin after serial dehydration steps, then sectioned at a thickness of 4 μm and stained with hematoxylin for 8 min and eosin for 5 min (HE staining). The histopathological changes of the ovaries in the sections were viewed using a Leica DM750 microscope (Germany).

### ELISA Analysis of Hormone and Pro-inflammatory Cytokines

The serum concentrations of hormones (T, E2, LH, and FSH) and pro-inflammatory cytokines (LPS, IL-18, IL-1β, IL-6 and TNF-α) were determined by ELISA kits according to the manufacturer’s instructions.

### Cell Culture and Treatment

RAW264.7 macrophages were purchased from FuHeng Biology Technology Co., Ltd. (Shanghai, China) and cultured in DMEM containing 10% FBS, as well as 1% penicillin-streptomycin at 37 C in an incubator with 5% CO_2_. In the study, cells were stimulated with LPS to simulate the inflammatory micro-environment in PCOS ovary. The concentration and time of LPS were determined by using NO assay kit as described previously. The viability of cells treated with different concentrations of SGD was detected by CCK-8 assay. The RAW264.7 macrophages were plated into 6-well plate at the concentration of 4 × 10^5^ cells per well and treated with LPS (100 ng/ml), SGD (10 mg/ml), LPS + SGD (1, 5, 10 mg/ml) at 37°C for 24°h, respectively. After that, the cells were harvested for RT-qPCR analysis.

### RT-qPCR

RT-qPCR was conducted as previously described (Hou et al., 2018). Total RNA was extracted using RNAprep pure Tissue/Cell Kit. Subsequently, the extracted RNA (approximately 1 μg) was used to synthesize first-strand cDNA by using the FastKing RT Kit. RT-qPCR reaction was subjected using a SuperReal PreMmix Color in the ABI StepOne Plus RT PCR system (United States), according to the manufacturer’s instructions. The primer sequences of Occludin, Claudin-1, ZO-1, TLR4, PI3K, Akt, p65, IL-18, IL-1β, IL-6, TNF-α and GAPDH were listed in Table1. The mRNA expressions of target genes were calculated using the 2^-∆∆Ct^ method and the results were shown as the relative amount normalized to GAPDH.

**TABLE 1:** List of primers for real-time PCR.

Source	Gene name	Forward primer (5′–3′)	Reverse primer (5′–3′)
	Occludin	GAC​CTT​GTC​CGT​GGA​TGA​CTT​CAG	ATC​AGC​AGC​AGC​CAT​GTA​CTC​TTC
	Claudin-1	ACT​GTG​GAT​GTC​CTG​CGT​TTC​G	GCA​GCA​GCC​CAG​CCA​GTA​AAG
	ZO-1	GAA​GGC​GGA​TGG​TGC​TAC​AAG​TG	AGG​CGA​AAG​GTA​AGG​GAC​TGG​AG
	TLR4	AGA​GAA​TCT​GGT​GGC​TGT​GGA​GAC	AAA​GGC​TTG​GGC​TTG​AAT​GGA​GTC
	PI3K	TCT​GGA​ATG​TGT​GGC​TGG​AGT​TTG	GGA​GGA​GGA​AGC​GGT​GGT​CTA​TC
Rat	Akt	CTC​AAC​AAC​TTC​TCA​GTG​GCA​CAA​TG	GCA​GGC​AGC​GGA​TGA​TGA​AGG
	p65	GAT​GGC​TTC​TAT​GAG​GCT​GAA​CTC​TG	CTT​GCT​CCA​GGT​CTC​GCT​TCT​TC
	TNF-α	AGC​ACG​GAA​AGC​ATG​ATC​CG	ACC​GAT​CAC​CCC​GAA​GTT​CA
	IL-1β	CCC​TTG​TCG​AGA​ATG​GGC​AG	GAC​CAG​AAT​GTG​CCA​CGG​TT
	IL-6	TCT​GCT​CTG​GTC​TTC​TGG​AGT​TCC	GTT​GGA​TGG​TCT​TGG​TCC​TTA​GCC
	IL-18	CAG​CCA​ACG​AAT​CCC​AGA​CC	AGA​TAG​GGT​CAC​AGC​CAG​TCC
	GAPDH	GTC​CAT​GCC​ATC​ACT​GCC​ACT​C	CGC​CTG​CTT​CAC​CAC​CTT​CTT​G
	TLR4	CCG​CTT​TCA​CCT​CTG​CCT​TCA​C	TGC​CGT​TTC​TTG​TTC​TTC​CTC​TGC
	PI3K	AGA​AAG​GTG​TGC​GGC​AGA​AGA​AG TAC​CAC​TAC​GGA​GCA​GGC​ATA​GC	TAC​CAC​TAC​GGA​GCA​GGC​ATA​GC
Mouse	Akt	AGA​GGC​AGG​AAG​AAG​AGA​CGA​TGG	GCA​GGA​CAC​GGT​TCT​CAG​TAA​GC
	p65	ACA​CCT​TCC​CAG​CAT​CCC​TCA​G	CTT​CCG​ACA​GCG​TGC​CTT​CC
	GAPDH	TCA​CCA​TCT​TCC​AGG​AGC​GAG​AC	TGA​GCC​CTT​CCA​CAA​TGC​CAA​AG

### Western Blot Analysis

The expression levels of related proteins were analyzed by western blot as described previously (Shao et al., 2020a). Briefly, protein samples were extracted using RIPA lysis buffer. After denaturation, the protein extracts were separated by electrophoresis on 10% SDS-PAGE for 1 h and then transferred to a polyvinylidene difluoride (PVDF) membrane (Merck Millipore Ltd. Co. Cork, IRL) for 3.0 h. Thereafter, the membrane was probed with the corresponding primary antibodies overnight at 4 C. The membrane was washed in TBST three times and incubated with HRP-conjugated secondary antibody at 25 C for 1 h. The immunoreactive signals were observed using a gel imaging system and the intensity of bands were analyzed by ImageJ software. The relative amount of target proteins were calculated by the gray value ratios of the protein to GAPDH and the relative phosphorylation was represented by the ratio of phosphorylated to total protein.

### 16S rRNA Gene Sequencing

Total DNA from the fecal sample was extracted using Mag-Bind Soil DNA Kit. Quality and purity of the DNA were monitored on 1% agarose gels. The V3-V4 region of 16S rRNA genes was amplified by specific primers with the barcode 341F (5′-CCTACGGGNGGCWGCAG-3′) and 806R (5′-GGACTACHVGGGTWTCTAAT -3′). The Cycling parameters were 98 C for 1 min, followed by 30 cycles of denaturation at 98 C for 10°s, annealing at 50 C for 30°s, and elongation at 72 C for 60°s with a final extension at 72 C for 5 min. After quantification and purification, the library was sequenced on an Illumina MiSeq platform with 250-bp paired-end reads. The sequence data was clustered into operational taxonomic units (OTUs) by UPARSE softwere with ≥97% similarity. α-diversity including Shannon and Simpson index were performed in QIIME (v1.8.0). β-diversity index principal co-ordinates analysis (PCoA) and Non-Metric Multi-Dimensional Scaling (NMDS) were performed by R packages to interpret the distance matrix. The relative abundance of major differences in bacteria taxa on the phylum and genus level was analyzed.

### Statistical Analysis

All data were expressed as means ± SEM and analyzed by GraphPad Prism 8.0 software. The Student’s t-test was used to compare the data between two independent groups, and the One-way ANOVA was used for Multiple-group comparisons. The correlation between specific gut microbiota and hormone levels, cytokines, tight junction proteins was tested by Spearman’s correlation test. A value of *p* < 0.05 is considered as statistically significant.

## Results

### SGD Alleviated the Symptoms in Letrozole-Induced PCOS Rats

**FIGURE 1:**
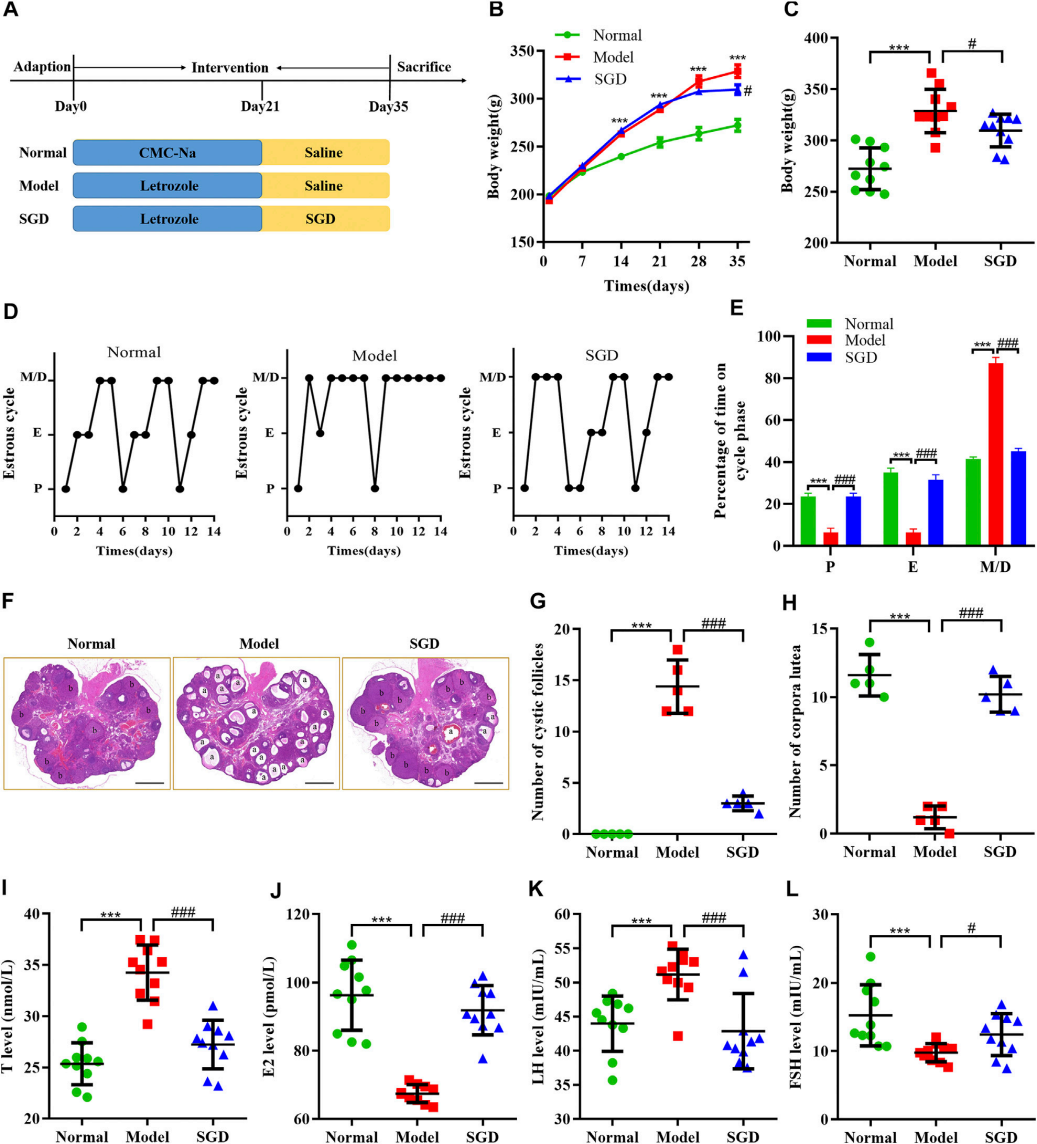
SGD alleviated the symptoms in Letrozole-induced PCOS rats. **(A)** Animal groups and treatments schedule. **(B,C)** Effect of SGD on body weight in PCOS rats (*n* = 10). **(D)** Representative estrous cycles. **(E)** Quantitative analysis of estrous cycles (*n* = 10). **(F)** HE staining of representative ovaries. “a” indicated the appearances of cystic follicles, “b” indicated the appearances of corpus luteum (magnification, ×10), Scale bar: 1 mm. **(G)** Quantitative analysis of cystic follicles (*n* = 5). **(H)** Quantitative analysis of corpus luteum (*n* = 5). **(I–L)** Serum levels of sex hormone indicators including testosterone. (T), 17β-estradiol (E_2_), luteinizing hormone (LH), and follicle stimulating hormone (FSH) were determined by using ELISA kits (*n* = 10). Data were presented as mean ± SEM. ***p < 0.001 vs. normal group, ^#^ *p* < 0.05 and ^###^ *p* < 0.001 vs. PCOS model group.

To verify the effect of SGD in treatment of PCOS, we investigated the body weight, estrous cycles, histological changes and serum hormone levels in three groups. Compared with the normal group, a higher weight gain was observed in PCOS rats, while treatment with SGD effectively reduced the increased body weight in PCOS rats (Figures 1B,C). Consistent with the previous studies, our data indicated that SGD could alleviate the obesity in PCOS rats (Shao et al., 2019). To further confirm the efficacy of SGD, the estrous cycle was recorded every day. As shown in Figures 1D,E PCOS rats showed disrupted estrous cycles with prolonged metaestrous and diestrous (M/D) phases as well as reduced proestrus and estrous phases. After 2°weeks of SGD treatment, the irregular estrous cycle was recovered to normal gradually. Next, H&E staining was investigated to observe morphological changes in rat ovary. In PCOS group, the numbers of cystic dilating follicles was increased and the corpus luteum disappeared, suggesting that the ovulatory function of PCOS rats was impaired. Notably, the administration of SGD ameliorated the damage effectively (Figures 1F–H). Furthermore, we assessed the regulative effect of SGD on serum hormone levels in PCOS rats. Compared with the normal group, the serum T and LH levels were significantly increased in PCOS rats, whereas the E2 and FSH levels were significantly decreased. As illustrated in Figures 1I–L, the abnormal hormone levels were significantly reversed by SGD treatment. Collectively, the above results indicated that SGD exhibits an excellent therapeutic effect in alleviating the endocrine abnormalities of PCOS rats.

### SGD Alleviated the Chronic Low-Grade Inflammation in PCOS Rats

**FIGURE 2:**
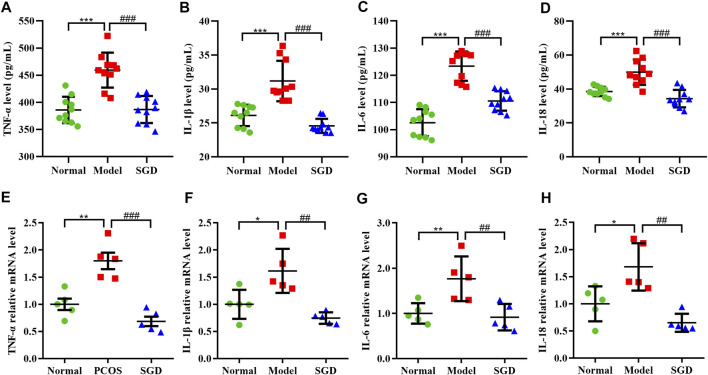
SGD alleviated the chronic low-grade inflammation in PCOS rats. **(A–D)** Serum levels of inflammatory cytokines including TNF-α, IL-1β, IL-6, and IL-18 were determined by using ELISA kits (*n* = 10). **(E–H)** The mRNA levels of inflammatory cytokines including TNF-α, IL-1β, IL-6, and IL-18 in rats ovarian tissues were quantitated by real-time PCR (*n* = 5). Data were presented as mean ± SEM. *p < 0.05, **p < 0.01, and ***p < 0.001 vs. normal group, ^##^ *p* < 0.01 and ^###^ *p* < 0.001 vs. PCOS model group.

Our previous study had demonstrated that PCOS is a chronic low-level inflammation state, which accompanied with the release of multiple cytokines. To determine the anti-inflammatory effect of SGD in PCOS rats, the levels of pro-inflammatory cytokines including IL-18, IL-1β, IL-6, and TNF-α were measured. The result showed the serum levels of these cytokines were increased in PCOS rats, whereas the increase was significantly suppressed by treatment with SGD (Figures 2A–D). Consistently, the ovarian mRNA expressions of TNF-α, IL-1β, IL-6, and IL-18 were significantly up-regulated in PCOS rats compared with the normal group; meanwhile, SGD treatment obviously attenuated the expressions (Figures 2E–H). Together, these results indicated that SGD alleviated the chronic low-grade inflammation in PCOS rats.

### SGD Treatment Modulated the Gut Microbiota Composition in PCOS Rats

**FIGURE 3:**
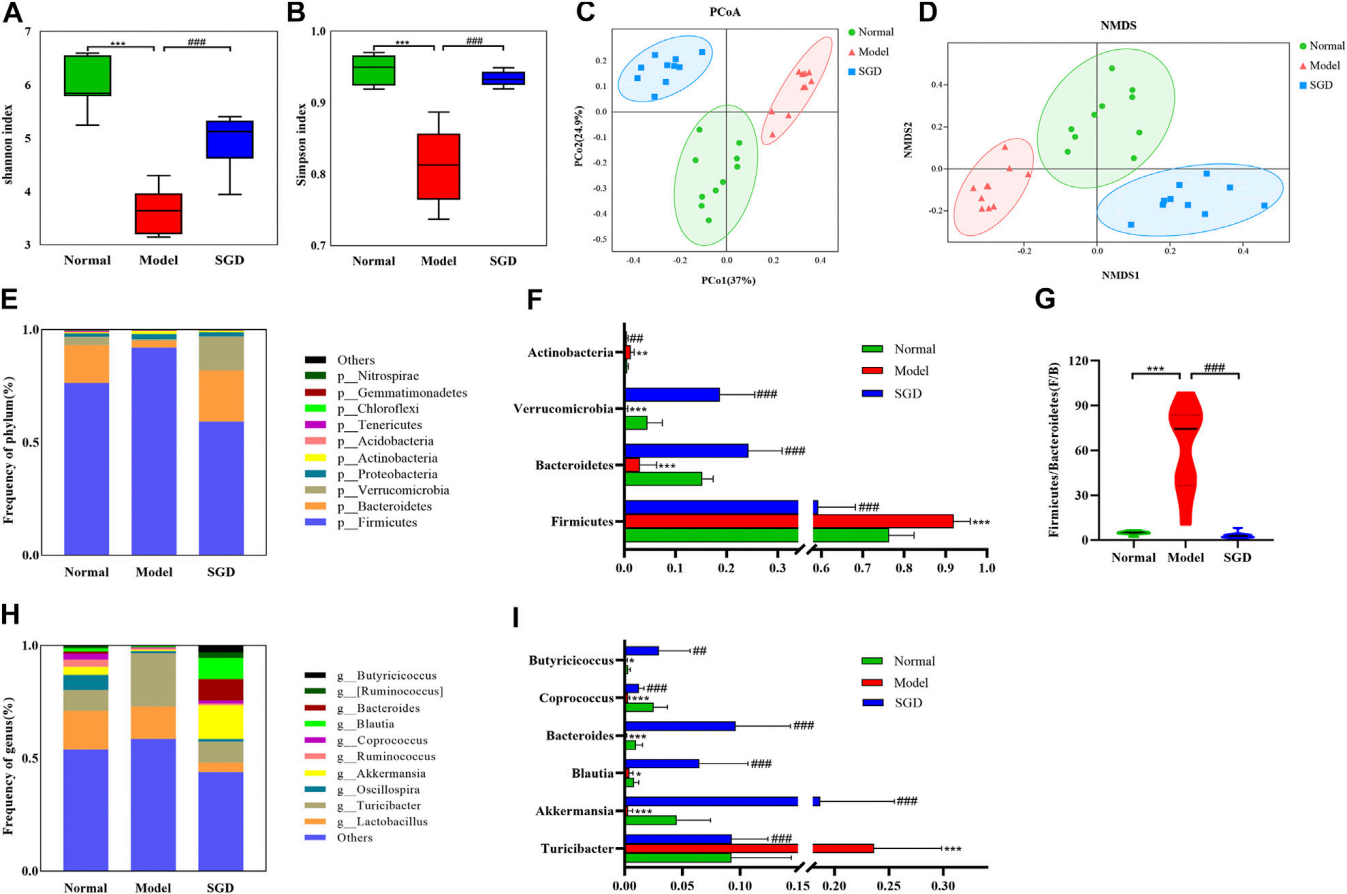
SGD treatment modulated the gut microbiota composition in PCOS rats. Shannon **(A)** and Simpson **(B)** diversity index. PCoA **(C)** and NMDS **(D)** plot of intestinal microbiota based on OTU level. **(E)** Column diagram of community composition at phylum level. **(F)** The relative abundances of *Firmicutes*, *Bacteroides*, *Verrucomicrobia*, and *Actinobacteria.* **(G)** *Firmicutes/Bacteroides* ratio. **(H)** Column diagram of community composition at genus level. **(I)** The relative abundances of *Turicibacter*, *Akkermansia*, *Blautia*, *Bacteroides*, *Coprococcus* and *Butyricicoccus* in three groups.

To address the role of gut microbiota in PCOS and the therapeutic mechanism of SGD, the 16S rRNA gene sequence analysis was performed. Across the 30 samples, an average of 64325 high-quality reads was obtained. In total, 2,814 operational taxonomic units (OTUs) were determined at 97% similarity level. Moreover, to examine the effect of SGD on the richness and diversity of gut microbiota, α diversity analysis was used. Shannon index and Simpson index showed that the intestinal microbial diversity of PCOS rats was significantly decreased compared with normal group, while SGD treatment reversed this change (Figures 3A,B). Furthermore, β diversity analysis was employed to identify the differences between microbial communities. PCoA and NMDS analysis showed that the intestinal microbiota clusters in the model group was visibly separated from that in the normal group. As shown in Figures 3C,D the closer distance between samples in the normal group and SGD group represented a smaller difference in the flora structure.

To further analyze the specific changes in gut bacteria, the relative abundance of microbial community was analyzed at the phylum and genus levels. In normal rats, the intestinal microflora was mainly composed of *Firmicutes* (76.36%), followed by *Bacteroides* (16.76%), *Verrucomicrobia* (3.63%), *Proteobacteria* (1.48%) and *Actinobacteria* (0.60%) at phylum levels, which were more than 98%. Among these, the abundance of *Firmicutes* and *Actinobacteria* were increased in the model group, while the administration of SGD reversed the alterations (Figures 3E,F). Meanwhile, PCOS rats displayed a significant decrease in abundance of *Bacteroidetes* and *Verrucomicrobia*, and SGD treatment enriched the relative abundance. Importantly, the ratio of *Firmicutes* to *Bacteroidetes* (F/B ratio), which together make up about 93% of gut microbiota, dramatically increased by more than 10 times in the model group compared that in the normal group (Figure 3G). Intriguingly, the ratio returned to normal level after treatment with SGD. The results indicated that SGD could improve the dysbacteriosis in rats with PCOS. At the genus level, *Lactobacillus* and *Turicibacter* were identified as the dominant bacteria in normal rats. Compared to the model group, SGD treatment significantly decreased the relative abundance of *Turicibacter* while increased those of *Akkermansia*, *Blautia*, *Bacteroides*, *Coprococcus* and *Butyricicoccus* (Figures 3H,I). Taken together, the results indicated that SGD treatment could effectively modulate the gut microbiota structure in PCOS rats.

### SGD Treatment Reduced the Serum LPS Level and Ameliorated the Intestinal Barrier Function in PCOS Rats

**FIGURE 4:**
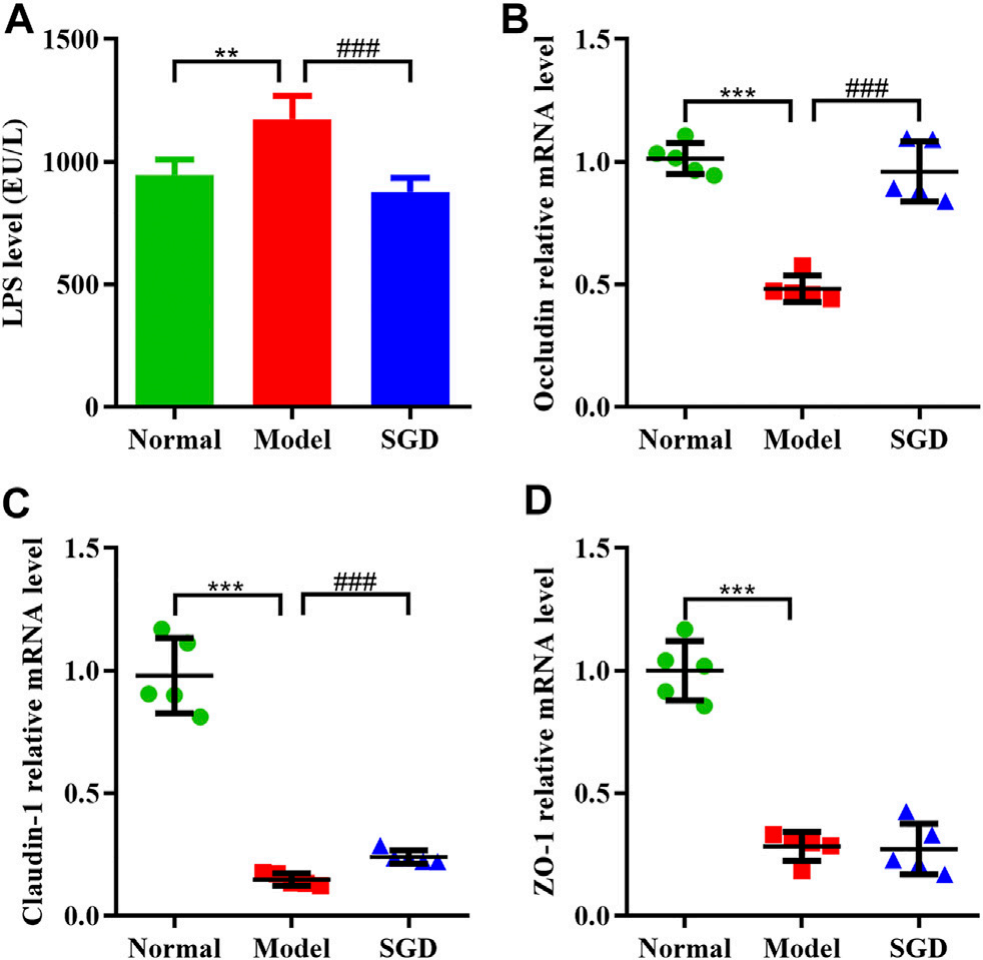
SGD treatment reduced the serum LPS level and ameliorated the intestinal barrier function in PCOS rats. **(A)** Serum levels of LPS were determined by using ELISA kits (*n* = 10). **(B–D)** The mRNA levels of tight junction proteins gene including occluding **(B)**, claudin-1 **(C)**, and ZO-1 **(D)** in rats ileum mucosa were quantitated by real-time PCR (*n* = 5). Data were presented as mean ± SEM. **p < 0.01 and ***p < 0.001 vs. normal group, ^###^ *p* < 0.001 vs. PCOS model group.

Compelling evidences indicated that an abnormal gut microbiome may lead to the damage of the intestinal wall and facilitate the transfer of LPS from intestine to blood. In this study, the serum LPS level of the model group was significantly higher than that of the normal group (*p* < 0.01). After SGD treatment, a restoration of the serum LPS level was observed (Figure 4A), which indicated that the bacterial translocation was weakened. Furthermore, to determine integrity of the gut barrier, the mRNA expressions of tight junction proteins including occludin, claudin-1, and ZO-1 in ileum mucosa were examined. Our data showed that the mRNA expressions of the three proteins were sharply decreased in PCOS rats compared with the normal group. Nevertheless, SGD could significantly up-regulate the expressions of occludin and claudin-1, but not ZO-1 (Figures 4B–D). Combined, our results suggested that SGD prevented the gut barrier damage in PCOS rats and reduced the endotoxin translocation.

### Correlation Analysis Among the Key Microbial Communities, Inflammatory Markers, Hormone Levels and Barrier-Function Parameters

**FIGURE 5:**
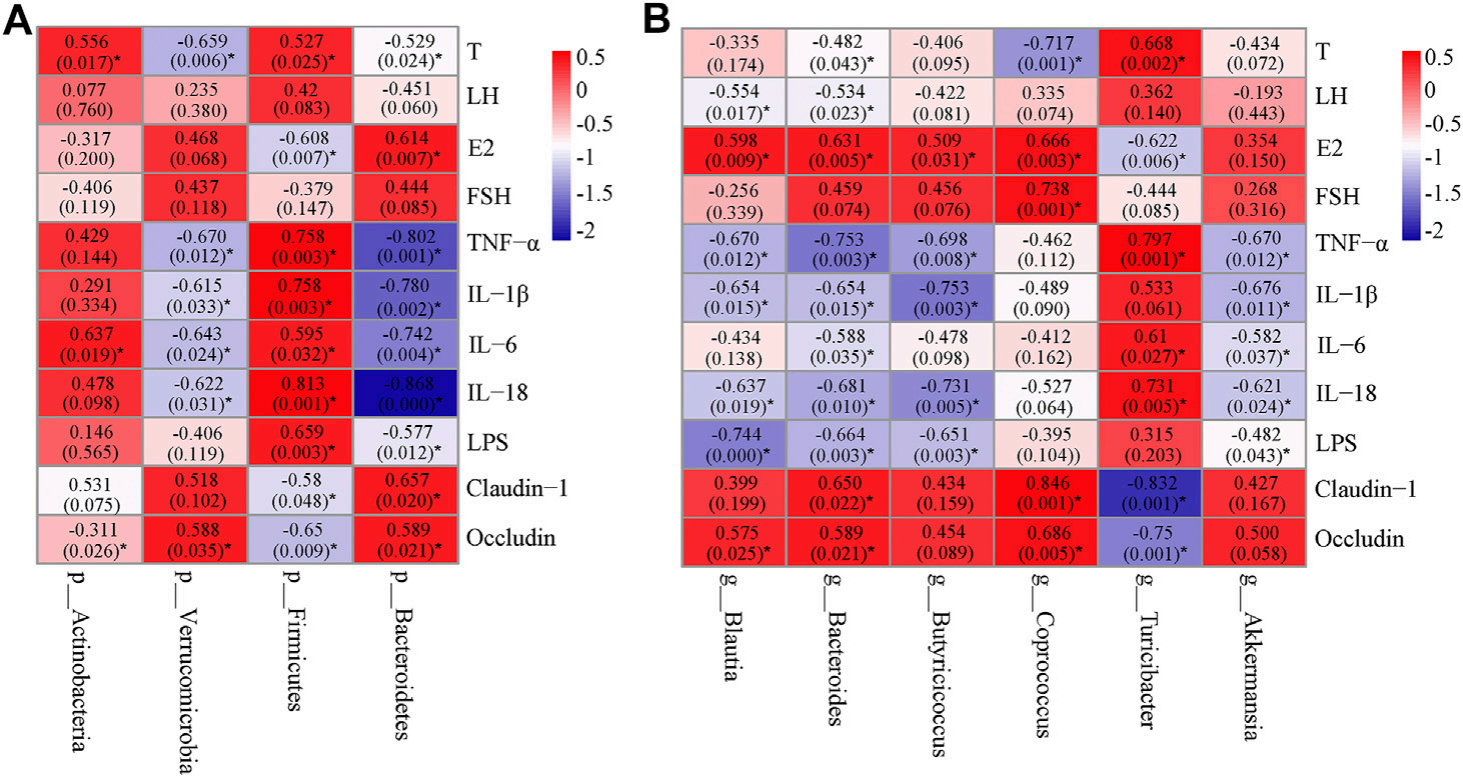
Heatmap of Spearman’s correlation between the gut microbiota [at phylum level **(A)** and at genus level **(B)**] and hormones, cytokines, LPS or tight junction proteins. The value of r represents the degree of correlation (0 > r > 1, positive correlation; −1<r < 0, negative correlation). Significant correlations were marked by *p < 0.05.

In order to find the key communities of gut microbiota associated with PCOS, Spearman’s correlation analysis was used to analyze the relationship between the specific gut microbiota and parameters related to PCOS. At the phylum level, *Firmicutes*, which was down-regulated by SGD, positively correlated with T, pro-inflammatory cytokines and LPS, but negatively correlated with E2, claudin-1, and occludin. Moreover, the relative abundance of *Bacteroidetes*, which were up-regulated by SGD, had negative correlation with T, pro-inflammatory cytokines and LPS, and positively correlated with E2, claudin-1, and occludin (Figure 5A). At the genus level, *Blautia* and *Bacteroides* showed a positive correlation with E2 and tight junction proteins, and negatively correlated with LH, inflammatory factors and LPS. In addition, *Butyricicoccus* and *Akkermansia* showed a significant negative correlation with the levels of TNF-α, IL-1β, IL-18 and LPS (Figure 5B). The results suggested that these bacteria played vital roles in the improvement effect of SGD on PCOS rats. The underlying mechanism may be related to the integrity of mucosal barrier, and then improve the inflammation state and hormone abnormalities.

### SGD Blunted TLR4/NF-κB Signaling Pathway in PCOS Rats and LPS-Stimulated RAW264.7 Cells

**FIGURE 6:**
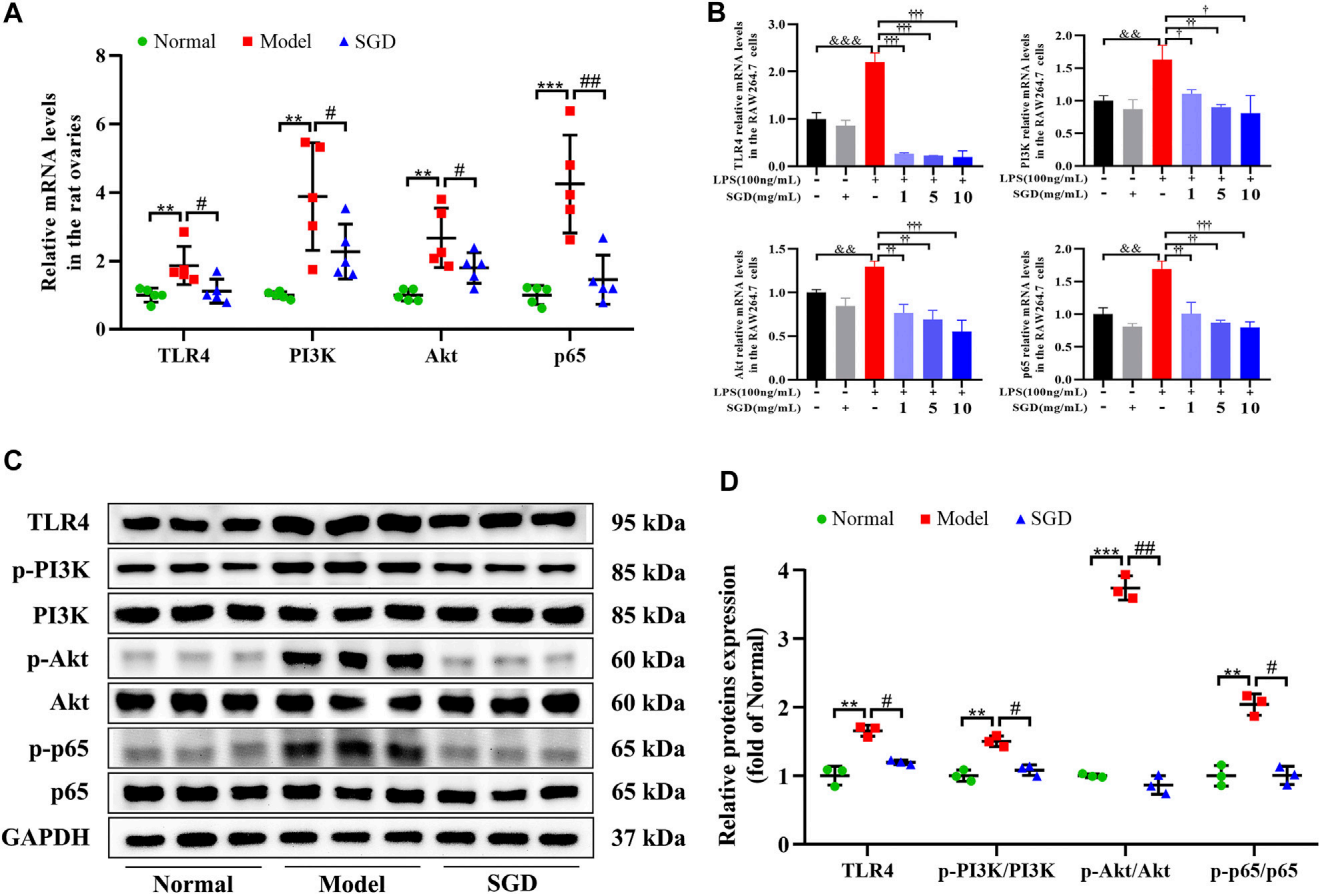
SGD blunted TLR4/NF-κB signaling pathway in PCOS rats and LPS-stimulated RAW264.7 cells. **(A)** The mRNA levels of TLR4, PI3K, Akt, and NF-κB p65 in rats ovarian tissues were quantitated by real-time PCR (*n* = 5). **(B)** The mRNA levels of TLR4, PI3K, Akt, and NF-κB p65 in RAW264.7 cells were quantitated by real-time PCR (*n* = 3). **(C,D)** The protein levels of TLR4, p-PI3K, PI3K, *p*-Akt, Akt, p-p65, and p65 in rats ovarian tissues were quantitated by Western blotting (*n* = 3). Data were presented as mean ± SEM. **p < 0.01 vs. normal group, #*p* < 0.05 and ##*p* < 0.01 vs. PCOS model group, &&*p* < 0.01 and &&&*p* < 0.001 vs. the untreated group, †*p* < 0.05, ††*p* < 0.01, and †††*p* < 0.001 vs. the LPS-stimulated group.

Emerging evidence revealed that the elevated circulatory levels of LPS could activate the inflammatory responses by inducing the activation of TLR4 and its downstream signaling. Hence, to give a better understanding on the molecule mechanism of SGD in treatment of PCOS, the gene and protein expressions of the TLR4/NF-κB signaling were analyzed. As illustrated in Figure 6A, the ovarian mRNA expressions of TLR4, PI3K, Akt, and NF-κB p65 were significantly up-regulated in PCOS rats compared with the normal group. However, treatment with SGD remarkably inhibited the increasing. Consistently, a significant increase in the ovarian protein levels of TLR4, as well as the relative expression ratios of p-PI3K to PI3K, p-Akt to Akt, and p-p65 to p65 was observed in the PCOS rats as compared to the normal group, while SGD treatment reversed the expressions (Figures 6C,D).

In addition, to further validate the molecule mechanism *in vitro*, the LPS-stimulated RAW264.7 cells were investigated. Before that, the viability of RAW264.7 cells treated with different concentrations of SGD was determined by CCK-8 assay kit, and 1, 5, 10 mg/ml of SGD was chosen for further testing (Supplementary Figure S2A). As shown in Figure 6B, SGD had no influence on the gene expressions of TLR4/NF-κB signaling in normal cells. Moreover, cells were stimulated with different concentrations of LPS (1, 10, 100, 1,000 and 10,000 ng/ml) for 3, 6, 12, 24 and 48 h. According to the NO production assay, the appropriate concentration of 100 ng/ml LPS for treating 24 h was chosen for the following experiments (Supplementary Figures S2B,C). After stimulated by LPS, the mRNA expressions of TLR4, PI3K, Akt, and NF-κB p65 were significantly elevated in cells, and these enhancements were reversed by SGD treatment. Together with the *in vivo* data, the results indicated that SGD could suppress inflammatory response by blunting TLR4/NF-κB signaling pathway.

## Discussion

PCOS is one of the most common endocrine disorders in women and often characterized by chronic low-grade inflammation as well as hormonal imbalance. In this work, the increased body weight and disrupted estrous cycle were observed in PCOS rats and reversed by SGD treatment. In addition, we also found that SGD could effectively decrease the number of cystic dilating follicles and regulate the abnormal steroid hormone levels of PCOS rats. Consistent with the previous studies (Shao et al., 2019), our results revealed that SGD exhibits an excellent therapeutic efficacy on PCOS.

During recent years, chronic low-grade inflammation has been considered as a potential contributor to the etiology of PCOS. The chronic pathological condition was reported to impair follicular growth and affect the ovulation process. For instance, more detailed studies showed that dysregulation of IL-1β and IL-18 contributed to ovulatory disruption and anovulation (Wang et al., 2020a). Our data also found increased number of cystic dilation follicles and irregular estrous cycles in PCOS rats. However, SGD treatment could effectively reduce the number of follicular cysts and the size of corpora lutea. These findings make us speculate that SGD may improve ovulation dysfunction by alleviating inflammation response in PCOS rats. The inflammation process, which acts as the vital role in the onset and development of PCOS, was characterized by a slight elevation in a host of pro-inflammatory cytokines. Consistently, in the current study, the serum and ovarian levels of the cytokines (including IL-18, IL-1β, IL-6, and TNF-α) were elevated in PCOS group; and SGD treatment could reverse the elevations. These results indicated that SGD could ameliorate both systemic and local inflammation in PCOS rats. However, the mechanism underlying how the chronic low-grade inflammation state in PCOS is induced still needs further research.

As we all known, the production of many pro-inflammatory factors in the host are induced by gut microbiota. For example, *Streptococcus* and its cell wall components have been shown to induce TNF-α and IL-1α secretion (Larsson et al., 1999). *Lactobacillus* has been shown to induce IL-6, IL-10, and TNF-α in human lymphocytes (Miettinen et al., 1998). Therefore, in order to better understand the key roles that gut microbiota play in the inflammation state of PCOS rats, we tested the abundance of gut microbiota. Our research showed that both α-diversity and β-diversity were significant altered in the pathological state of PCOS. At the phylum level, *Bacteroidetes* and *Firmicutes* are the dominant bacteria in the three groups. Several studies have reported that F/B ratio, which was regarded as the marker of dysbacteriosis, have positive association with enhanced inflammatory cytokines expressions (Chu et al., 2019). Consistently, in the current study, this ratio was dramatically increased (12.52 folds) in the model group and reversed by the supplementation of SGD. The F/B ratio was positively correlated with the levels of TNF-α, IL-1β, IL-18 and IL-6. The lower F/B ratio is considered favorable for the amelioration of inflammation. Yu et al. had reported that the alterations of intestinal microbiota, which was characterized by the decreased F/B ratio, could alleviate inflammatory infiltration (Yu et al., 2017). Moreover, our data also showed that the ratio was positively correlated with the levels of LPS and testosterone, and negatively correlated with tight junction protein as well as estradiol. The disordered sex hormone levels have been characterized as the most important clinical symptoms in patients with PCOS. Thus, we speculated that F/B ratio may serve as a potential marker/risk indicator for PCOS. *Verrucomicrobia*, the environmental microorganisms, was decreased in PCOS rats and sharply elevated after SGD treatment. Although the significance of Verrucomicrobia remains inconclusive, members of this phylum are thought to be associated with chronic inflammatory diseases (Dubourg et al., 2013; Depommier et al., 2019). As the only representative genus of this phylum in human gut, *Akkermansia*, has attracted emerging interest for its health benefits. In our study, the abundance of *Akkermansia* was decreased in PCOS rats compared with normal rats, and sharply elevated after the administration of SGD. It is reported that *Akkermansia* is a mucin-degrading bacterium and can reduce circulating LPS levels (Yang et al., 2019; Wang et al., 2020b). Likewise, our results also showed the abundances of *Akkermansia* were negatively correlated with the serum levels of LPS and the expressions of proinflammatory cytokines (IL-6, IL-18, TNF-α and IL-1β). Recent studies indicated that *Akkermansia* treatment could reduce the expressions of TNF-α and IL-1β, which consisted with our results. *Akkermansia* could inhibit macrophage functions, chemokines secretion and thus improve metabolic profile (Wang et al., 2020b). According to a recent clinical trial, supplementation of *Akkermansia* could reduce the levels of the inflammation markers and reinforced gut barrier function in human volunteers (Barcena et al., 2019). However, in our study, there were no significant correlations between *Akkermansia* and the tight junction proteins. The inconsistent results require further experimental explanation. At the genus level, we found that *Turicibacter* and *Lactobacillus* were dominant in both PCOS rats and normal rats by examination of fecal samples. *Lactobacillus*, known as probiotics, was slightly decreased in model group. Meanwhlie, the abundance of *Turicibacter* was increased in PCOS rats and significant declined in SGD group. Although the role of *Turicibacter* as pathogens still controversial, our results were in accordance with some studies, which demonstrated increased abundance of *Turicibacter* was positively correlated with proinflammatory cytokines (Wu et al., 2020). Taken together, the results of 16S rRNA gene sequence analysis revealed the SGD ameliorated the inflammation state in PCOS rats via remodeling gut microbiota to some extent.

To further clarify the correlation between the specific gut microbiota and inflammation parameters related to PCOS, we made a correlation analysis between the key microbial communities and inflammatory markers. The results showed that the relative abundance of *p_Firmicutes* and *g_Turicibacter*, which were considered as pro-inflammatory gut bacteria, were positively correlated with the levels of IL-6, IL-18 and TNF-α. On the contrary, the relative abundance of *p_Bacteroidetes*, *p_Verrucomicrobia, g_Bacteroides* and *g_Akkermansia* had negative correlation with the pro-inflammatory cytokines, and were supposed as anti-inflammatory gut bacteria. More importantly, we found that the abundance of anti-inflammatory gut bacteria was negatively correlated with the serum level of testosterone and positively correlated with E2, which was opposite of the association at pro-inflammatory gut bacteria. We all know that the inflammatory process can lead to the hormonal imbalance in PCOS (Shao et al., 2019). Therefore, we speculated that SGD could improve the inflammation state and hormone abnormalities in PCOS rats by enriching the abundance of anti-inflammatory gut bacteria and reducing the abundance of pro-inflammatory gut bacteria.

LPS, a major product from Gram-negative bacteria, was reported to participate in the pathogenesis of several metabolic derangements. In this study, a significant increase in the abundance of LPS-producing pathogens (such as *Proteobateria*) was observed in PCOS rats. Subsequently, an elevated serum level of LPS in PCOS rats was observed, which indicated an increased entry of bacterial endotoxin into systemic circulation. As reported, the increased circulating LPS is deemed as a risk factor for PCOS. Duleba et al. demonstrated that LPS profoundly increased the expression of key genes in androgen synthesis and contributed to hyperandrogenemia (Banaszewska et al., 2020). To some extent, this helps explain the reason why the administration of SGD could ameliorate the hyperandrogenism in PCOS rats by reducing the LPS level.

Nowadays, it is becoming recognized that the impairment of gut barrier (referred to as “leaky gut”) was closely linked to many chronic inflammatory diseases including PCOS (Guo et al., 2016). The “leaky gut” allows the gut microbiota-derived endotoxin LPS into systemic circulation and leads to a systemic state of immune activation. In this work, to examine the integrity of the intestinal barrier, the expressions of tight junction proteins were analyzed. As a result, the expressions of occludin and claudin1 were remarkably decreased in model group, and recovered after SGD treatment, suggesting that the prescription could protect the mucosal barrier in PCOS rats.

The communication between gut microbes and their host is based on the bacterial metabolites. In addition to LPS, other metabolites such as short-chain fatty acids (SCFA) and bile acid (BA) were also related to the development of PCOS. SCFA was considered as an important energy source for the maintenance of the intestinal epithelium (Chen et al., 2019). In the present study, after SGD administration, the abundance of SCFA-producing bacteria (e.g., *Butyricicoccus, Coprococcus, Akkermansia* and *Blautia*) was notably increased compared with model group. To further verify the gut-protective effect of these bacteria, the correlation analysis was conducted. Consistently, the abundances of the reported probiotics-*Coprococcus* and *Blautia* were positively correlated with TJ proteins. Furthermore, the abundance of *Butyricicoccus, Akkermansia* and *Blautia*, was negatively correlated with the expressions of proinflammatory cytokines. These genera, which were reported to produce butyrate and acetic acid, play vital roles in suppressing inflammatory response (Zhang et al., 2019b). Taking these results into consideration, we speculated that the anti-inflammatory effect of SGD may be related to the SCFA-producing bacteria, and the mechanism needs further investigation. As reported, BAs is an important signaling molecule that regulates inflammatory response in PCOS (Qi et al., 2019; Li et al., 2021). Our present study found that the abundance of *Bacteroides*, which is known as the lithocholic acid (LCA)-producing bacteria, was decreased in PCOS rats compared with normal rats, and dramatically increased after the administration of SGD. As we know, LCA is a secondary BA and can activate multiple transcription factors [such as farnesoid X receptor (FXR)] and BA receptors (such as Takeda G-protein coupled receptor 5 (TGR5)] (Zhang et al., 2019a; Hui et al., 2020; Liang et al., 2020). FXR can antagonize inflammation by negatively regulating the NF-κB signaling and reducing the expression of inflammatory cytokines (Shao et al., 2020a). These findings may well explain our results that the negative correlation between the abundance of *Bacteroides* and the levels of proinflammatory cytokines.

To further explain the molecule mechanism of SGD in the microbiota-modulated effect of PCOS, the expressions of the TLR4/NF-κB signaling (an important downstream pathway of LPS) were examined. The present data show that SGD could down-regulating expressions of TLR4 and its downstream signaling molecules PI3K, Akt and NF-κB p65 in PCOS rats. Moreover, the administration of SGD could also reduce the levels of pro-inflammatory cytokines such as TNF-α, IL-6, IL-18 and IL-1β. These results together with the previous researches lead us to conjecture that SGD may regulate the communication between gut and ovary by suppressing the TLR4-mediated LPS response in PCOS rats. As reported, macrophages were the main contributor for the inflammatory cytokines production in ovary (Choi et al., 2020). Clinically, macrophage infiltration was thought to be closely related to the chronic inflammation state in PCOS patients (Alanbay et al., 2012). Therefore, to further verify the molecule mechanism, LPS-stimulated RAW264.7 macrophages were conducted to simulate the inflammatory micro-environment in the PCOS ovary. In line with the results from *in vivo* study, we observed that SGD could markedly suppress the key genes expressions of TLR4/NF-κB signaling pathway in LPS-induced RAW264.7 cells. The activated TLR4/NF-κB signaling could trigger a succession of downstream inflammatory cascades such as the activation of NOD-like receptor protein 3 (NLRP3) inflammasome, caspase-1 and C-reactive protein (CRP). These factors were proved to participate in different PCOS phenotypes, such as insulin resistance, abdominal obesity and abnormal lipid metabolism (Barrea et al., 2019; Herman et al., 2020). In conjunction with the present results, we suggested that the role of SGD on improving the inflammation state in PCOS was achieved by suppressing the TLR4/NF-κB signaling pathway.

In conclusion, the current study demonstrated that SGD could improve the chronic low-grade inflammation state in PCOS rats via remodeling the gut microbiota and reducing the endotoxin translocation. The underlying mechanism may be related to protect the gut barrier function and suppress TLR4/NF-κB signaling pathway. These findings provided a scientific basis for promoting the treatment of PCOS with SGD.

## Data Availability

The datasets presented in this study can be found in online repositories. The names of the repository/repositories and accession numbers can be found below: BioProject ID PRJNA712931.

## Glossary

ANOVA: analysis of variance
BCA: bicinchoninic acid
CCK-8: cell counting kit-8
CRP: C-reactive protein
DMEM: Dulbecco’s modified Eagle’s medium
E2: 17beta-estradiol
ELISA: enzyme-linked immunosorbent assay
FBS: fetal bovine serum
FSH: follicle stimulating hormone
FXR: farnesoid X receptor
GAPDH: glyceraldehyde-3-phosphate dehydrogenase;
HE: hematoxylin-eosin
HRP: horseradish peroxidase
IL-: interleukin-
LCA: lithocholic acid
LH: luteinizing hormone
LPS: lipopolysaccharide;
NF-κB: nuclear factor-κB
NMDS: Non-Metric Multi-Dimensional Scaling
NO: nitric oxide
NLRP3: NOD-like receptor protein 3
p-: phosphorylated-
PCoA: principal co-ordinates analysis
PCOS: Polycystic ovary syndrome
PI3K: phosphatidylinositol-3-kinase
PVDF: polyvinylidene fluoride
SD: Sprague Dawley
SGD: Shaoyao-Gancao Decoction
T: testosterone
TBST: Tris Buffered Saline with Tween
TGR5: Takeda G-protein coupled receptor 5
TLR4: Toll-like receptor 4
TNF-α: tumor necrosis factor-α

## References

[B1] Alanbay I. Ercan C. M. Sakinci M. Coksuer H. Ozturk M. Tapan S. (2012). A Macrophage Activation Marker Chitotriosidase in Women with PCOS: Does Low-Grade Chronic Inflammation in PCOS Relate to PCOS Itself or Obesity? Arch. Gynecol. Obstet. 286, 1065–1071. 10.1007/s00404-012-2425-0 22718099

[B2] Anagnostis P. Tarlatzis B. C. Kauffman R. P. (2018). Polycystic Ovarian Syndrome (PCOS): Long-Term Metabolic Consequences. Metabolism 86, 33–43. 10.1016/j.metabol.2017.09.016 29024702

[B3] Banaszewska B. Siakowska M. Chudzicka-Strugala I. Chang R. J. Pawelczyk L. Zwozdziak B. (2020). Elevation of Markers of Endotoxemia in Women with Polycystic Ovary Syndrome. Hum. Reprod. 35, 2303–2311. 10.1093/humrep/deaa194 32869098

[B4] Bárcena C. Valdés-Mas R. Mayoral P. Garabaya C. Durand S. Rodríguez F. (2019). Healthspan and Lifespan Extension by Fecal Microbiota Transplantation into Progeroid Mice. Nat. Med. 25, 1234–1242. 10.1038/s41591-019-0504-5 31332389

[B5] Barrea L. Arnone A. Annunziata G. Muscogiuri G. Laudisio D. Salzano C. (2019). Adherence to the Mediterranean Diet, Dietary Patterns and Body Composition in Women with Polycystic Ovary Syndrome (PCOS). Nutrients 11, 2278. 10.3390/nu11102278 PMC6836220 31547562

[B6] Chen R. Xu Y. Wu P. Zhou H. Lasanajak Y. Fang Y. (2019). Transplantation of Fecal Microbiota Rich in Short Chain Fatty Acids and Butyric Acid Treat Cerebral Ischemic Stroke by Regulating Gut Microbiota. Pharmacol. Res. 148, 104403. 10.1016/j.phrs.2019.104403 31425750

[B7] Choi J. H. Jang M. Kim E.-J. Lee M. J. Park K. S. Kim S.-H. (2020). Korean Red Ginseng Alleviates Dehydroepiandrosterone-Induced Polycystic Ovarian Syndrome in Rats via its Antiinflammatory and Antioxidant Activities. J. Ginseng Res. 44, 790–798. 10.1016/j.jgr.2019.08.007 33192122 PMC7655494

[B8] Chu W. Zhai J. Xu J. Li S. Li W. Chen Z. J. (2019). Continuous Light-Induced PCOS-like Changes in Reproduction, Metabolism, and Gut Microbiota in Sprague-Dawley Rats. Front. Microbiol. 10, 3145. 10.3389/fmicb.2019.03145 32038578 PMC6990112

[B9] de Medeiros S. F. de Medeiros M. a. S. Santos N. S. Barbosa B. B. Yamamoto M. M. W. (2018). Combined Oral Contraceptive Effects on Low-Grade Chronic Inflammatory Mediators in Women with Polycystic Ovary Syndrome: A Systematic Review and Meta-Analysis. Int. J. Inflam 2018, 9591509. 10.1155/2018/9591509 30595838 PMC6286752

[B10] Depommier C. Everard A. Druart C. Plovier H. Van Hul M. Vieira-Silva S. (2019). Supplementation with Akkermansia Muciniphila in Overweight and Obese Human Volunteers: a Proof-Of-Concept Exploratory Study. Nat. Med. 25, 1096–1103. 10.1038/s41591-019-0495-2 31263284 PMC6699990

[B11] Dubourg G. Lagier J.-C. Armougom F. Robert C. Audoly G. Papazian L. (2013). High-level Colonisation of the Human Gut by Verrucomicrobia Following Broad-Spectrum Antibiotic Treatment. Int. J. Antimicrob. Agents 41, 149–155. 10.1016/j.ijantimicag.2012.10.012 23294932

[B12] Eyupoglu N. D. Ergunay K. Acikgoz A. Akyon Y. Yilmaz E. Yildiz B. O. (2020). Gut Microbiota and Oral Contraceptive Use in Overweight and Obese Patients with Polycystic Ovary Syndrome. J. Clin. Endocrinol. Metab. 105, dgaa600. 10.1210/clinem/dgaa600 32860695

[B13] Guo Y. Qi Y. Yang X. Zhao L. Wen S. Liu Y. (2016). Association between Polycystic Ovary Syndrome and Gut Microbiota. PLoS One 11, e0153196. 10.1371/journal.pone.0153196 27093642 PMC4836746

[B14] Heinrich M. Appendino G. Efferth T. Fürst R. Izzo A. A. Kayser O. (2020). Best Practice in Research - Overcoming Common Challenges in Phytopharmacological Research. J. Ethnopharmacol. 246, 112230. 10.1016/j.jep.2019.112230 31526860

[B15] Herman R. Jensterle M. Janez A. Goricar K. Dolzan V. (2020). Genetic Variability in Antioxidative and Inflammatory Pathways Modifies the Risk for PCOS and Influences Metabolic Profile of the Syndrome. Metabolites 10, 439. 10.3390/metabo10110439 PMC7692942 33138337

[B16] Hou R.-g. Fan L. Liu J.-j. Cheng Y. Chang Z.-p. Wu B. (2018). Bile Acid Malabsorption Is Associated with Diarrhea in Acute Phase of Colitis. Can. J. Physiol. Pharmacol. 96, 1328–1336. 10.1139/cjpp-2018-0017 30383974

[B17] Hui S. Huang L. Wang X. Zhu X. Zhou M. Chen M. (2020). Capsaicin Improves Glucose Homeostasis by Enhancing Glucagon‐like Peptide‐1 Secretion through the Regulation of Bile Acid Metabolism via the Remodeling of the Gut Microbiota in Male Mice. FASEB j. 34 (6), 8558–8573. 10.1096/fj.201902618rr 32359008

[B18] Insenser M. Murri M. Del Campo R. Martínez-García M. Á. Fernández-Durán E. Escobar-Morreale H. F. (2018). Gut Microbiota and the Polycystic Ovary Syndrome: Influence of Sex, Sex Hormones, and Obesity. J. Clin. Endocrinol. Metab. 103, 2552–2562. 10.1210/jc.2017-02799 29897462

[B19] Larsson B. M. Larsson K. Malmberg P. Palmberg L. (1999). Gram Positive Bacteria Induce IL-6 and IL-8 Production in Human Alveolar Macrophages and Epithelial Cells. Inflammation 23, 217–230. 10.1023/a:1020269802315 10392756

[B20] Li T. Zhang T. Gao H. Liu R. Gu M. Yang Y. (2021). Tempol Ameliorates Polycystic Ovary Syndrome through Attenuating Intestinal Oxidative Stress and Modulating of Gut Microbiota Composition-Serum Metabolites Interaction. Redox Biol. 41, 101886. 10.1016/j.redox.2021.101886 33592539 PMC7896192

[B21] Liang L.-m. Zhou J.-j. Xu F. Liu P.-h. Qin L. Liu L. (2020). Diabetes Downregulates Peptide Transporter 1 in the Rat Jejunum: Possible Involvement of Cholate-Induced FXR Activation. Acta Pharmacol. Sin 41 (11), 1465–1475. 10.1038/s41401-020-0408-4 32341465 PMC7656584

[B22] Liu J. j. Cheng Y. Shao Y. y. Chang Z. p. Guo Y. t. Feng X. j. (2019). Comparative Pharmacokinetics and Metabolites Study of Seven Major Bioactive Components of Shaoyao‐Gancao Decoction in Normal and Polycystic Ovary Syndrome Rats by Ultra-high Pressure Liquid Chromatography with Tandem Mass Spectrometry. J. Sep. Sci. 42, 2534–2549. 10.1002/jssc.201900002 31144455

[B23] Liu L. Li Y. H. Niu Y. B. Sun Y. Guo Z. J. Li Q. (2010). An Apple Oligogalactan Prevents against Inflammation and Carcinogenesis by Targeting LPS/TLR4/NF- B Pathway in a Mouse Model of Colitis-Associated Colon Cancer. Carcinogenesis 31, 1822–1832. 10.1093/carcin/bgq070 20400476

[B24] Luo S. Wen R. Wang Q. Zhao Z. Nong F. Fu Y. (2019). Rhubarb Peony Decoction Ameliorates Ulcerative Colitis in Mice by Regulating Gut Microbiota to Restoring Th17/Treg Balance. J. Ethnopharmacol. 231, 39–49. 10.1016/j.jep.2018.08.033 30170079

[B25] Miettinen M. Matikainen S. Vuopio-Varkila J. Pirhonen J. Varkila K. Kurimoto M. (1998). Lactobacilli and Streptococci Induce Interleukin-12 (IL-12), IL-18, and Gamma Interferon Production in Human Peripheral Blood Mononuclear Cells. Infect. Immun. 66, 6058–6062. 10.1128/iai.66.12.6058-6062.1998 9826398 PMC108774

[B26] Qi X. Yun C. Sun L. Xia J. Wu Q. Wang Y. (2019). Gut Microbiota-Bile Acid-Interleukin-22 axis Orchestrates Polycystic Ovary Syndrome. Nat. Med. 25, 1225–1233. 10.1038/s41591-019-0509-0 31332392 PMC7376369

[B27] Rostamtabar M. Esmaeilzadeh S. Tourani M. Rahmani A. Baee M. Shirafkan F. (2021). Pathophysiological Roles of Chronic Low‐grade Inflammation Mediators in Polycystic Ovary Syndrome. J. Cel Physiol 236, 824–838. 10.1002/jcp.29912 32617971

[B28] Shao Y. Y. Chang Z. P. Cheng Y. Wang X. C. Zhang J. P. Feng X. J. (2019). Shaoyao-Gancao Decoction Alleviated Hyperandrogenism in a Letrozole-Induced Rat Model of Polycystic Ovary Syndrome by Inhibition of NF-kappaB Activation. Biosci. Rep. 39, BSR20181877. 10.1042/BSR20181877 30573529 PMC6328870

[B29] Shao Y. Y. Guo Y. Feng X. J. Liu J. J. Chang Z. P. Deng G. F. (2020a). Oridonin Attenuates TNBS-Induced Post-inflammatory Irritable Bowel Syndrome via PXR/NF-kappaB Signaling. Inflammation 30, 645–658. 10.1007/s10753-020-01364-0 33125572

[B30] Shao Y. Y. Guo Y. T. Gao J. P. Liu J. J. Chang Z. P. Feng X. J. (2020b). Shaoyao-Gancao Decoction Relieves Visceral Hyperalgesia in TNBS-Induced Postinflammatory Irritable Bowel Syndrome via Inactivating Transient Receptor Potential Vanilloid Type 1 and Reducing Serotonin Synthesis. Evid. Based Complement. Alternat Med. 2020, 7830280. 10.1155/2020/7830280 33123210 PMC7584960

[B31] Torres P. J. Siakowska M. Banaszewska B. Pawelczyk L. Duleba A. J. Kelley S. T. (2018). Gut Microbial Diversity in Women with Polycystic Ovary Syndrome Correlates with Hyperandrogenism. J. Clin. Endocrinol. Metab. 103, 1502–1511. 10.1210/jc.2017-02153 29370410 PMC6276580

[B32] Tremellen K. Pearce K. (2012). Dysbiosis of Gut Microbiota (DOGMA) - A Novel Theory for the Development of Polycystic Ovarian Syndrome. Med. Hypotheses 79, 104–112. 10.1016/j.mehy.2012.04.016 22543078

[B33] Wang D. Weng Y. Zhang Y. Wang R. Wang T. Zhou J. (2020a). Exposure to Hyperandrogen Drives Ovarian Dysfunction and Fibrosis by Activating the NLRP3 Inflammasome in Mice. Sci. Total Environ. 745, 141049. 10.1016/j.scitotenv.2020.141049 32758727

[B34] Wang L. Tang L. Feng Y. Zhao S. Han M. Zhang C. (2020b). A Purified Membrane Protein from Akkermansia Muciniphila or the Pasteurised Bacterium Blunts Colitis Associated Tumourigenesis by Modulation of CD8+ T Cells in Mice. Gut 69, 1988–1997. 10.1136/gutjnl-2019-320105 32169907 PMC7569398

[B35] Wang T. Sha L. Li Y. Zhu L. Wang Z. Li K. (2020c). Dietary Alpha-Linolenic Acid-Rich Flaxseed Oil Exerts Beneficial Effects on Polycystic Ovary Syndrome through Sex Steroid Hormones-Microbiota-Inflammation *Axis* in Rats. Front. Endocrinol. (Lausanne) 11, 284. 10.3389/fendo.2020.00284 32670195 PMC7326049

[B36] Wu M. Yang S. Wang S. Cao Y. Zhao R. Li X. (2020). Effect of Berberine on Atherosclerosis and Gut Microbiota Modulation and Their Correlation in High-Fat Diet-Fed ApoE-/- Mice. Front. Pharmacol. 11, 223. 10.3389/fphar.2020.00223 32231564 PMC7083141

[B37] Yang Y. Zhong Z. Wang B. Xia X. Yao W. Huang L. (2019). Early-life High-Fat Diet-Induced Obesity Programs Hippocampal Development and Cognitive Functions via Regulation of Gut Commensal Akkermansia Muciniphila. Neuropsychopharmacol. 44, 2054–2064. 10.1038/s41386-019-0437-1 PMC6897910 31207607

[B38] Yu L. Zhao X. Cheng M. Yang G. Wang B. Liu H. (2017). *Saccharomyces* Boulardii Administration Changes Gut Microbiota and Attenuates D-Galactosamine-Induced Liver Injury. Scientific Rep. 7 (1), 1–7. 10.1038/s41598-017-01271-9 PMC5430957 28465509

[B39] Zhang B. Shen S. Gu T. Hong T. Liu J. Sun J. (2019a). Increased Circulating Conjugated Primary Bile Acids Are Associated with Hyperandrogenism in Women with Polycystic Ovary Syndrome. J. Steroid Biochem. *Mol. Biol.* 189, 171–175. 10.1016/j.jsbmb.2019.03.005 30849463

[B40] Zhang W. Xu J.-H. Yu T. Chen Q.-K. (2019b). Effects of Berberine and Metformin on Intestinal Inflammation and Gut Microbiome Composition in Db/db Mice. Biomed. Pharmacother. 118, 109131. 10.1016/j.biopha.2019.109131 31545226

[B41] Zhu Y. Li Y. Liu M. Hu X. Zhu H. (2020). Guizhi Fuling Wan, Chinese Herbal Medicine, Ameliorates Insulin Sensitivity in PCOS Model Rats with Insulin Resistance via Remodeling Intestinal Homeostasis. Front. Endocrinol. (Lausanne) 11, 575. 10.3389/fendo.2020.00575 32973686 PMC7482315

