# Supplementary material for: Shaoyao-Gancao Decoction Ameliorates the Inflammation State in Polycystic Ovary Syndrome Rats via Remodeling Gut Microbiota and Suppressing the TLR4/NF-κB Pathway
Source: Front Pharmacol. 2021 May 13;12:670054. doi: 10.3389/fphar.2021.670054 (PMC8155368; doi:10.3389/fphar.2021.670054)
Supplement: Supplementary file 1 [file DataSheet1.PDF]

Supplementary materials

Part I: The quantity control of SGD.

Supplementary Fig. 1. HPLC-QTRAP-MS/MS profiles to detect SGD. 1: Oxypaeoniflorin (retention time=1.40 min); 2: Albiflorin (1.98 min); 3: Paeoniflorin (2.52 min); 4: Liquiritin (3.86 min); 5: Liquiritigenin (6.49 min); 6: Glycyrrhizin (7.09 min); 7: Glycyrrhetinic acid (8.37 min); 8: genistein (6.71 min)

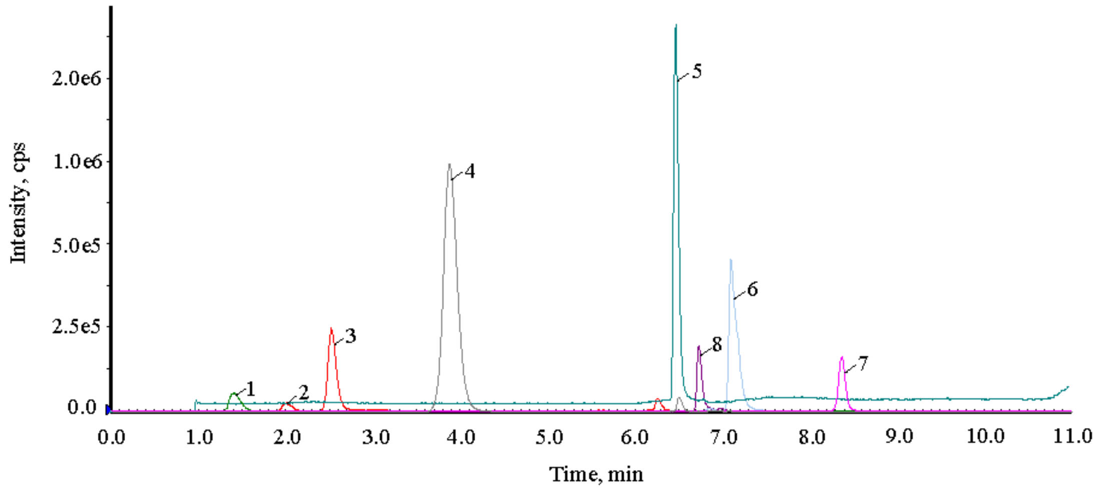

Supplementary Table 1. The regression equations of seven analytes in SGD.

Analyte	Regression equation	R^2
Oxypaeoniflorin	y = 0.0017x - 0.0188	0.9969
Albiflorin	y = 0.0001x - 0.0004	0.9962
Paeoniflorin	y = 0.0003x - 0.0059	0.9923
Liquiritin	y = 0.0121x - 0.0023	0.9998
Liquiritigenin	y = 0.014x - 0.0014	0.9993
Glycyrrhizin	y = 0.0001x + 0.0008	0.9977
Glycyrrhetinic acid	y = 0.001x - 0.0178	0.9991

Part II: The SGD and LPS concentrations detection in vitro experiments

Supplementary Fig. 2. (A) RAW264.7 cells were exposed to the different

concentrations of SGD for 24 h, and cell viability was measured using the CCK-8 assay. The concentration of NO produced by RAW264.7 cells treated with different concentrations of LPS for 24 hours (B) and different times of LPS (100 ng/mL) (C).

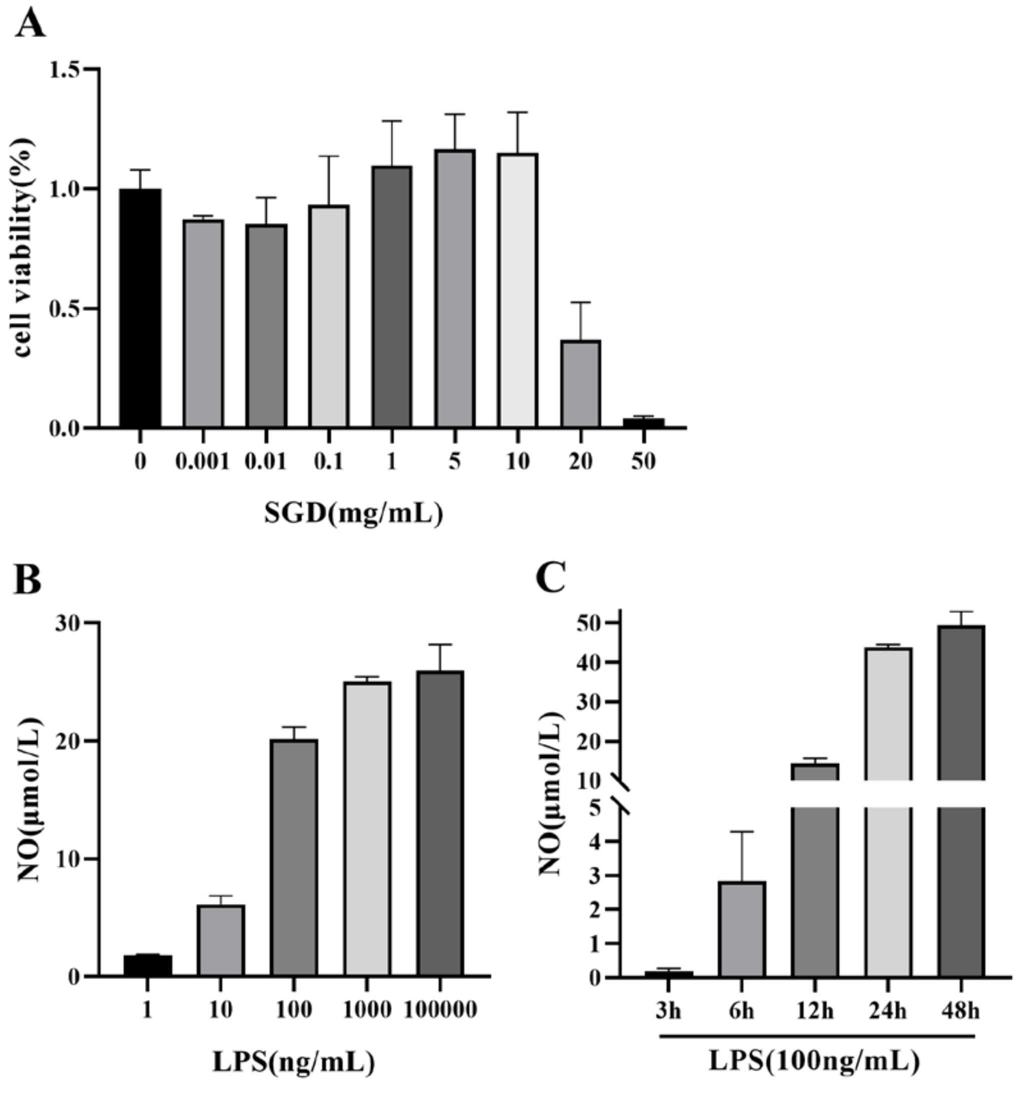

Part III: SGD reduced the relative abundance of *Proteobacteria* in PCOS rats.

Supplementary Fig. 3. The relative abundance of *Proteobacteria* in three groups (n=10). Data were presented as mean ± SEM. $^{**}P$<0.01 versus normal group, $^{\#\#}P$<0.01 versus PCOS model group.

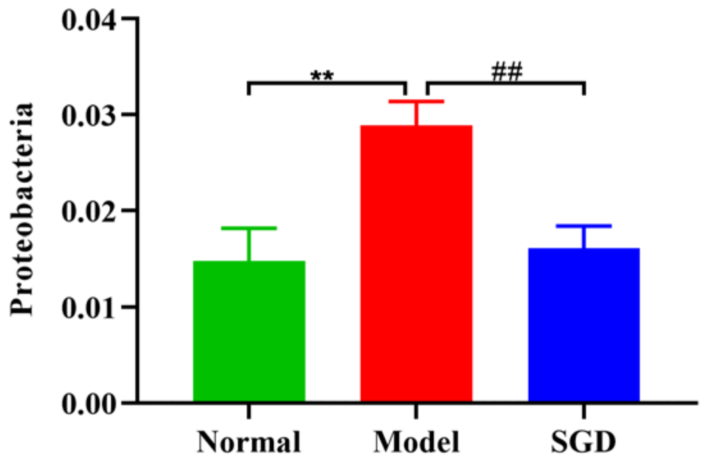

Proteobacteria
0.04
0.03
0.02
0.01
0.00
**
##
Normal
Model
SGD
